# Bioinspired Heparin Nanosponge Prepared by Photo-crosslinking for Controlled Release of Growth Factors

**DOI:** 10.1038/s41598-017-14040-5

**Published:** 2017-10-30

**Authors:** Won Il Choi, Abhishek Sahu, Cristian Vilos, Nazila Kamaly, Seong-Min Jo, Jin Hyung Lee, Giyoong Tae

**Affiliations:** 1 Center for Convergence Bioceramic Materials, Convergence R&D Division, Korea Institute of Ceramic Engineering and Technology, 202, Osongsaengmyeong 1-ro, Osong-eup, Heungdeok-gu, Cheongju, Chungbuk 28160 Republic of Korea; 2 School of Materials Science and Engineering, Gwangju Institute of Science and Technology, 123 Cheomdan-gwagiro, Buk-gu, Gwangju 61005 Republic of Korea; 3 Universidad Andres Bello, Laboratory of Nanomedicine and Targeted Delivery, Center for Integrative Medicine and Innovative Science, Faculty of Medicine, Center for Bioinformatics and Integrative Biology, Faculty of Biological Sciences, Santiago, 8370071 Chile; 4 Center for the Development of Nanoscience and Nanotechnology, CEDENNA, 9170124 Santiago, Chile; 5 Technical University of Denmark, Department of Micro and Nanotechnology, DTU Nanotech, Bioinspired Nanomaterials Lab, 2800 Kgs, Lyngby, Denmark; 6 Max Planck Institute for Polymer Research, Ackermannweg 10, 55128 Mainz, Germany

## Abstract

Growth factors have great therapeutic potential for various disease therapy and tissue engineering applications. However, their clinical efficacy is hampered by low bioavailability, rapid degradation *in vivo* and non-specific biodistribution. Nanoparticle based delivery systems are being evaluated to overcome these limitations. Herein, we have developed a thermosensitive heparin nanosponge (Hep-NS) by a one step photopolymerization reaction between diacrylated pluronic and thiolated heparin molecules. The amount of heparin in Hep-NS was precisely controlled by varying the heparin amount in the reaction feed. Hep-NS with varying amounts of heparin showed similar size and shape properties, though surface charge decreased with an increase in the amount of heparin conjugation. The anticoagulant activity of the Hep-NS decreased by 65% compared to free heparin, however the Hep-NS retained their growth factor binding ability. Four different growth factors, bFGF, VEGF, BMP-2, and HGF were successfully encapsulated into Hep-NS. *In vitro* studies showed sustained release of all the growth factors for almost 60 days and the rate of release was directly dependent on the amount of heparin in Hep-NS. The released growth factors retained their bioactivity as assessed by a cell proliferation assay. This heparin nanosponge is therefore a promising nanocarrier for the loading and controlled release of growth factors.

## Introduction

Growth factors are a class of soluble protein, which act as signalling molecules to modulate cell proliferation, differentiation, and survival. These biomolecules are essential to stimulate tissue growth and repair^1,2^. They have very short half-lives but at the same time, they can exert their influence at very low concentrations^2,3^. Growth factors have great potential as biotherapeutics in many clinical applications, especially in the clinical restoration of a variety of tissue defects resulting from trauma, such as degenerative disease and oncological resection^4^. Furthermore, growth factors are essential for the clinical success of tissue engineering, which focuses on the repair or replacement of the damaged tissues in our body^1–3^. To mimic the natural tissue regeneration process via an engineered scaffold, multiple growth factors should be delivered at an optimized ratio with a controlled rate of release^1,4^.

Advances in protein purification and recombinant DNA technology have made it possible to obtain many natural and recombinant growth factors for clinical applications. However, only a few growth factors have been approved and commercialized for therapeutic use in humans. The low rate of clinical success is mainly due to their inherent instability in solution, self-aggregation, poor bioavailability, short *in vivo* half-life, and challenges associated with *in vivo* delivery^1,3,5^. Generally, growth factors are delivered as either a bolus injection into the site of disease or by systemic administration^1,5^. It is difficult to administer them over long periods of time in a bioactive form at physiological doses. Thus, to attain a therapeutic effect *in vivo*, requires administration of extremely high doses, which often results in unwanted side effects such as hypotension, retinopathy, or progression of malignant tumors due to off-target effects at distant sites^1,5^.

Therefore, to improve their clinical success and reduce any unwanted side effects there is a considerable need to optimize growth-factor delivery so that their local concentration in the target tissue is sustained over time, and their bioactivity is retained with minimal impact on normal organs^3,5^. Since the last decade, many efforts have been made to develop controlled release systems for growth factor delivery^6^. Micro/nano-particles, hydrogels, and porous 3D scaffolds are a few examples of different systems used for growth factor delivery^3,5,6^. Their release profiles are controlled by adjusting the physiochemical properties of the vehicle, such as porosity, degree of cross-linking and degradation rate. Delivery systems have also been designed to produce differential release profiles, distinct spatiotemporal control release and stimuli responsive release^2,3,5,6^. However, these efforts of incorporating growth factors within a sustained release vehicle have been only partially successful due to low bioactivity and new materials are needed to further improve the efficacy of the released growth factors.

Heparin is a natural, highly sulfated anionic polysaccharide, clinically used as an anticoagulant^7^. Heparin has specific interactions with a variety of proteins that have heparin-binding domains, including most of the growth factors^8,9^. Therefore, several heparin containing systems have been developed for the sustained release of growth factors^9,10^. Biodegradable poly(D,L-lactic-co-glycolic acid) (PLGA) microspheres and nanoparticles functionalized with heparin were widely used for the delivery of several growth factors, such as basic fibroblast growth factor (bFGF), and vascular endothelial growth factor (VEGF), amongst others^11–13^. Heparin was also conjugated to various implantable 3D scaffolds such as collagen, alginate, and chitosan for controlled delivery of growth factors^14–16^. Nanoparticles prepared by physical self-assembly of Pluronic-conjugated heparin showed controlled release of the loaded drug owing to the presence of heparin. However, the application of this self-assembled nanosystem might have limitations such as low loading efficiency, instability during lyophilization/resuspension for storage, and low stability in the *in vivo* environment^17^.

Nanosponge refers to a class of nanomaterials with nanoporous structure and superior absorption/complexation properties^18^. A variety of ‘nanosponges’ based on organic/polymeric or inorganic materials have been reported in the past^18–21^. In addition to being porous, a nanosponge can also refer to a particle system with a high volumetric expansion property^22^. In this study, we have developed photo-crosslinked thermosensitive heparin nanosponges as a chemically stable vehicle for the sustained release of various growth factors for prolonged effects. The term ‘nanosponge’ was used to emphasize the fact that this nanoparticle system has a large volume expansion property in response to temperature change, which is useful for efficient loading of biomolecules such as growth factors, without using any organic solvents and harsh conditions^22^. The heparin nanosponges were characterized as a new type of carrier for loading and controlled release of four different yet important growth factors; bFGF, VEGF, bone morphogenic protein 2 (BMP-2), and hepatocyte growth factor (HGF). The amount of heparin was varied within the nanosponge and the release of the growth factors was monitored. Furthermore, the bioactivity of the released growth factor was also analyzed.

## Results and Discussion

### Preparation and characterization of heparin nanosponges (Hep-NS)

**Figure 1:**
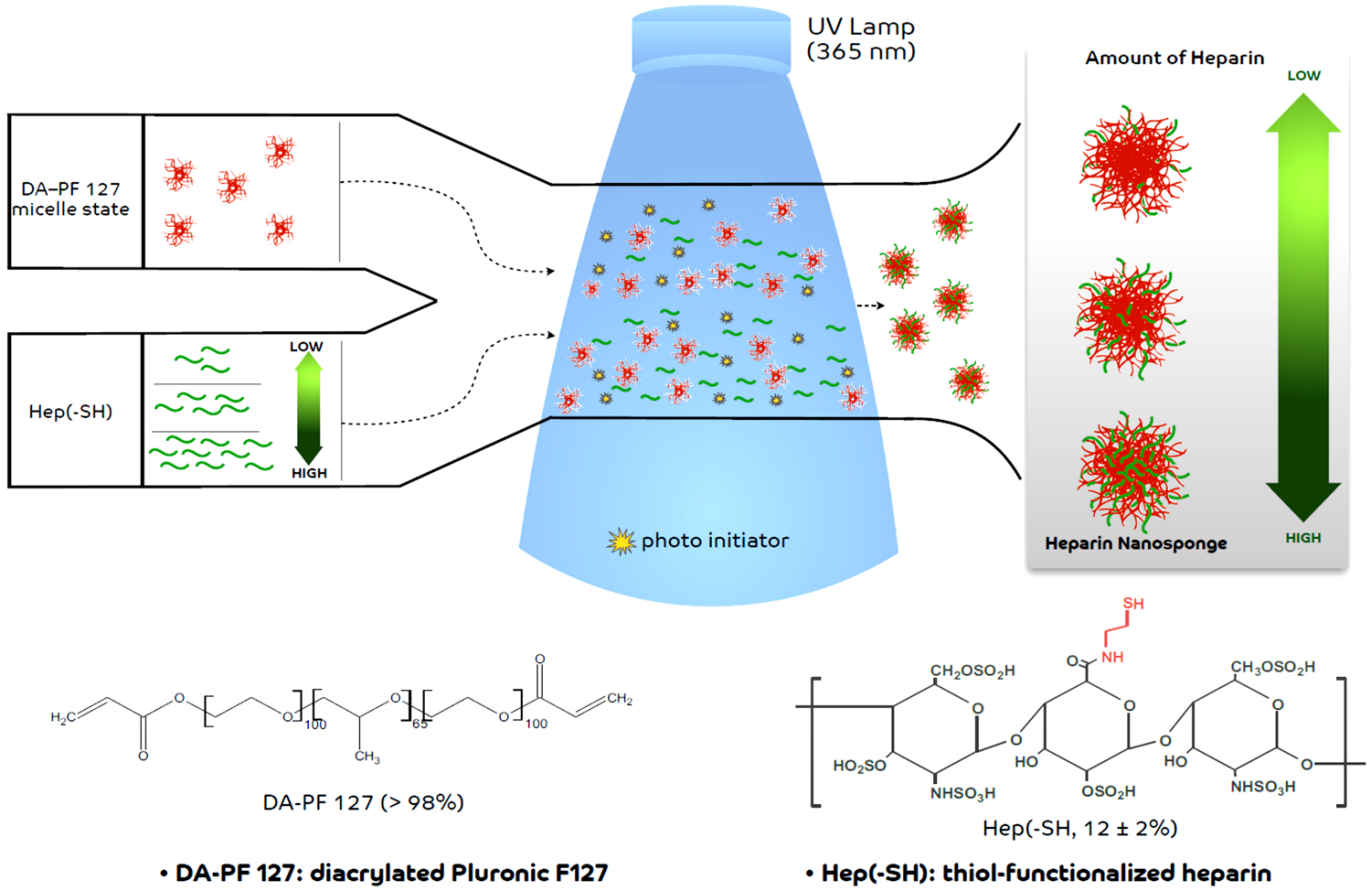
Schematic description of the preparation of photo-crosslinked heparin nanosponges (Hep-NS).

The heparin nanosponges were prepared by a UV light initiated thiol-ene reaction between thiolated heparin (Hep-SH) and diacrylated Pluronic F127 (DA-PF 127) as shown in Fig. 1 ^23,24^. The thiol-ene reaction resulted in a quick conjugation between thiolated biomolecules and acrylated polymers based on a radical polymerization process as previously reported^23,25,26^. This simple one-step photo-crosslinking method resulted in the formation of relatively mono-dispersed nanosponges with high conjugation yields. The amount of heparin in the nanosponges could be tuned from 3 wt % to 24 wt % by simply varying the Hep-SH concentration in the reaction feed. Increasing the heparin amount beyond 24 wt% resulted in uncontrollable aggregates as assessed by nanoparticle size (data not shown).

**Figure 2:**
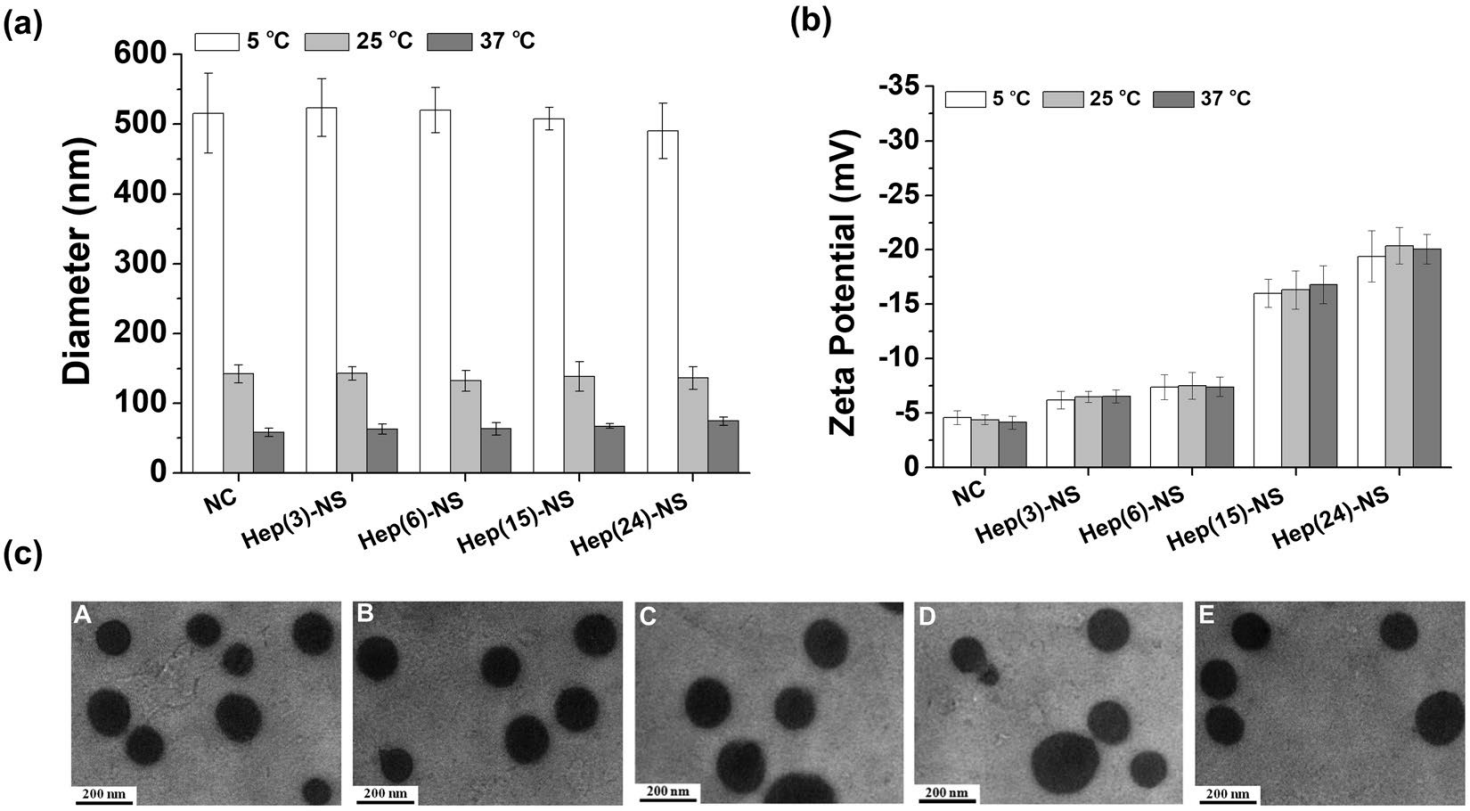
(**a**) Hydrodynamic diameters and (**b**) surface charges (zeta-potential) of nano-carrier (NC) and heparin nanosponge (Hep-NS) at 5 °C, 25 °C, and 37 °C. Hep(X)-NS denotes the amount of heparin (X wt%) in relation to the nanosponge. (**c**) TEM images of (A) NC, (B) Hep(3)-NS, (C) Hep(6)-NS, (D) Hep(15)-NS, and (E) Hep(24)-NS. Scale bar: 200 nm.

The heparin nanosponges (Hep-NS) showed similar hydrodynamic sizes and thermo-sensitive properties compared to those of the bare Pluronic-based nano-carrier (NC) with size changes from 516 ± 57 nm at 5 °C to 58 ± 6 nm at 37 °C (Fig. 2a). The heparin amount in the nanosponge had no effect on the particle size or temperature dependent volume change. However, the surface charge of the nanosponges gradually decreased from −4.1 ± 0.6 mV to −29.1 ± 1.5 mV as the heparin amount increased, confirming that highly negatively charged heparin was present on the surface of the nanosponges (Fig. 2b). All the different Hep-NS showed similar size and morphology by TEM imaging (Fig. 2c), confirming that the heparin conjugation did not affect their morphology or their size. In addition, we tried to observe the detailed morphology of the heparin nanosponge under high magnification at different temperatures (5, 25, or 37 °C). Temperature dependent size changes of the Hep(24)-NS was observed by TEM imaging (Supplementary Figure S1), however the detailed nanoporous structure could not be clearly observed, even at high magnification. Use of TEM for imaging of organic/ polymeric materials has intrinsic limitations, where previous reports have also had limited success in observing nanoporous structures by TEM^18,19^. In our previous study, we investigated the release of gold nanoparticles of 5 or 10 nm in size from Pluronic nano-carriers at 4 °C and 37 °C to analyze their nano-porosity^24^. As a result, the effective pore size of the nano-carriers at 37 °C was estimated to be between 5 to 10 nm. Therefore, the heparin nanosponges have a similar nanoporous structure since the Hep-NS show similar physico-chemical properties to our previously developed Pluronic nano-carriers (NC)^24^.

### Anticoagulant activity of Hep-NS

**Figure 3:**
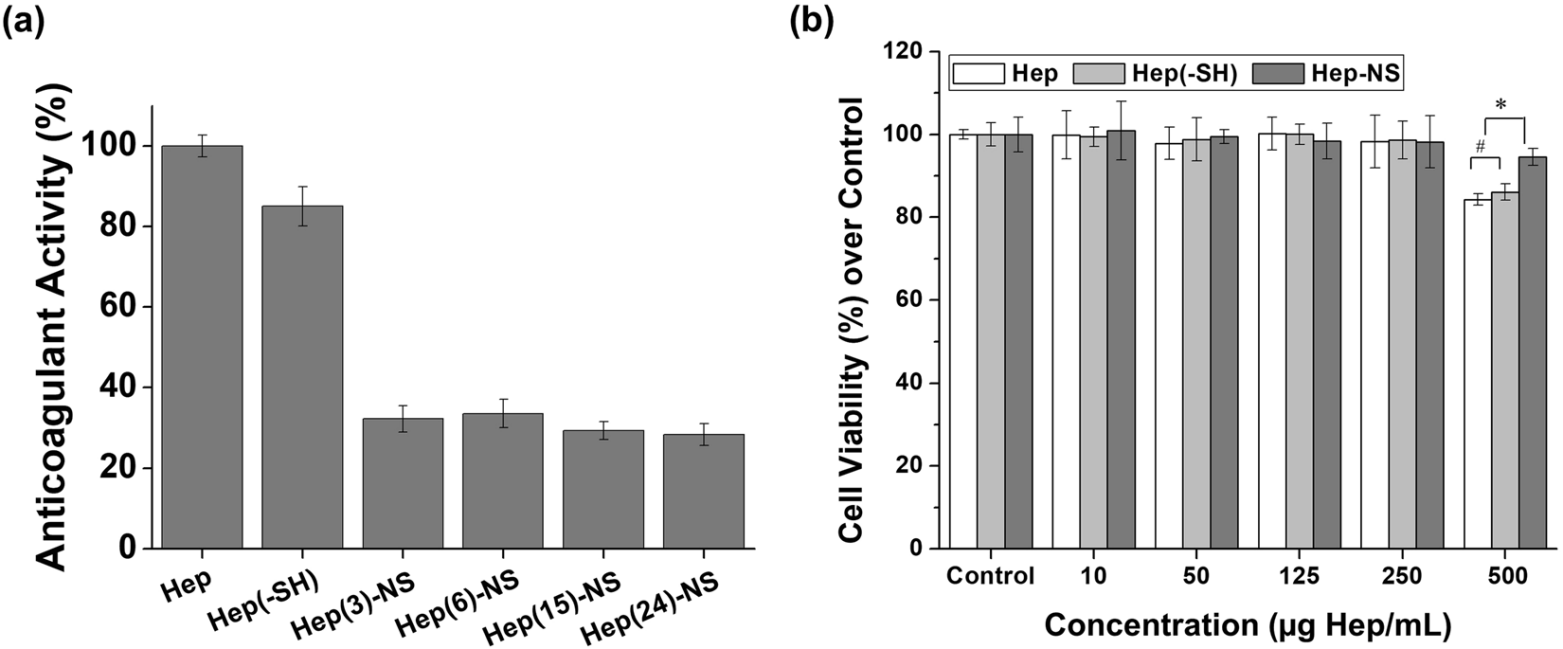
(**a**) Anticoagulant activity of heparin, thiolated heparin, and heparin nanosponge (Hep-NS) measured by its binding affinity to AT-III. (b) Cytotoxicity of heparin, thiolated heparin, and heparin nanosponge (Hep(24)-NS) in Balb/c3T3 fibroblast cells. (n = 3, ^#^p > 0.05 and *p < 0.05).

The anticoagulation activity of the free heparin or the heparin nanosponges was measured by a FXa inhibition assay. As shown in Fig. 3a, thiol modified heparin (Hep-SH) showed a slight decrease in anticoagulant activity compared to the intact heparin, suggesting a reduced binding affinity of Hep-SH towards AT-III. However, significant anticoagulant activity was retained, which compares well with previous reports showing that the sulfate group of heparin is mainly responsible for its anticoagulant activity^27,28^. On the other hand, all the heparin nanosponges demonstrated almost 65% reduction in anticoagulant activity compared to the intact heparin. Unmodified, fully anticoagulant heparin can exhibit side effects such as heparin-induced thrombocytopenia or hemorrhagic complications (excessive bleeding), which is a major concern for *in vivo* applications of heparin based materials^9,29–31^. Therefore, this significant reduction in anticoagulant activity of Hep-NS can be beneficial for application in non-thrombotic indications such as growth factor delivery.

### *In vitro* serum stability and cytotoxicity of Hep-NS

The *in vitro* serum stability of the Hep-NS was characterized in a serum-containing cell culture media at 37 °C under shaking (100 rpm). The hydrodynamic sizes and polydispersity of all nanosponges remained without any significant change for up to 2 weeks (Supplementary Table S1), suggesting that the Hep-NS can be stable without forming large aggregates in an *in vivo* environment. Importantly, the chemically cross-linked Hep-NS were lyophilized without any cryo-protectants, and subsequently could be re-suspended in aqueous media without forming aggregates, implying excellent stability and ease of use post freeze/thaw for storage and further applications^24,32^.

Furthermore, the acute cytotoxic effect of free heparin or heparin nanosponges at various heparin concentrations was characterized by the CCK-8 assay on Balb/c3T3 fibroblast cells. As shown in Fig. 3b, the intact heparin, thiolated heparin, and heparin (24 wt%)-nanosponge did not affect metabolic activity of the cells up to 250 μg/mL (based on heparin amount). Additionally, at 500 μg/mL, free heparin showed some noticeable cytotoxicity, whereas the nanosponges remained nontoxic, suggesting lower apoptosis induction by Hep-NS than free heparin molecules^33,34^. Thus, the Hep-NS have the potential to minimize limitations associated with the safety and side effects of heparin for *in vivo* applications.

### *In vitro* release profiles of growth factors from Hep-NS

**Figure 4:**
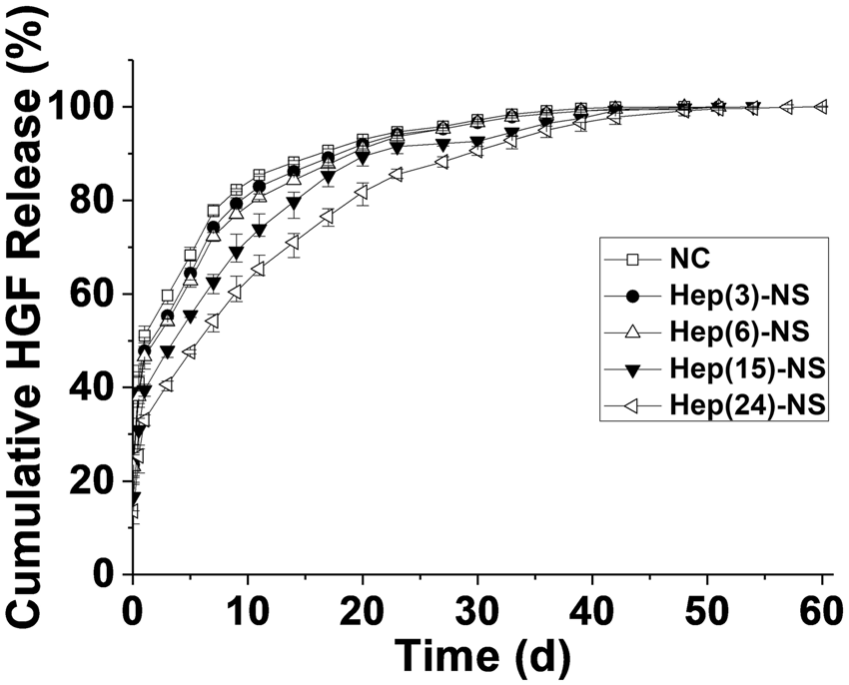
*In vitro* release profiles of HGF from the nano-carrier (NC) and heparin nanosponge (Hep-NS) with various amounts of heparin.

In order to investigate the release effect of growth factors in relation to the amount of heparin within the nanosponges, hepatocyte growth factor (HGF), which is a heparin binding growth factor was selected. This growth factor could be efficiently loaded into the nanosponges with over 90% retention, and the sizes and surface charges of the nanosponges were not altered by loading the growth factor, suggesting effective shielding of the growth factors within the nanosponges, similar to previously developed Pluronic-based nano-carriers by us^24,28,35^. The release patterns of the growth factor from the nanosponges in a physiological buffer (PBS, pH 7.4) was directly dependent on the heparin concentration in the Hep-NS (Fig. 4). Higher amounts of heparin (24 wt%) in the Hep-NS resulted in a much more sustained release of the growth factor over a period of one month compared to other nanosponges with lower amounts of heparin.

**Figure 5:**
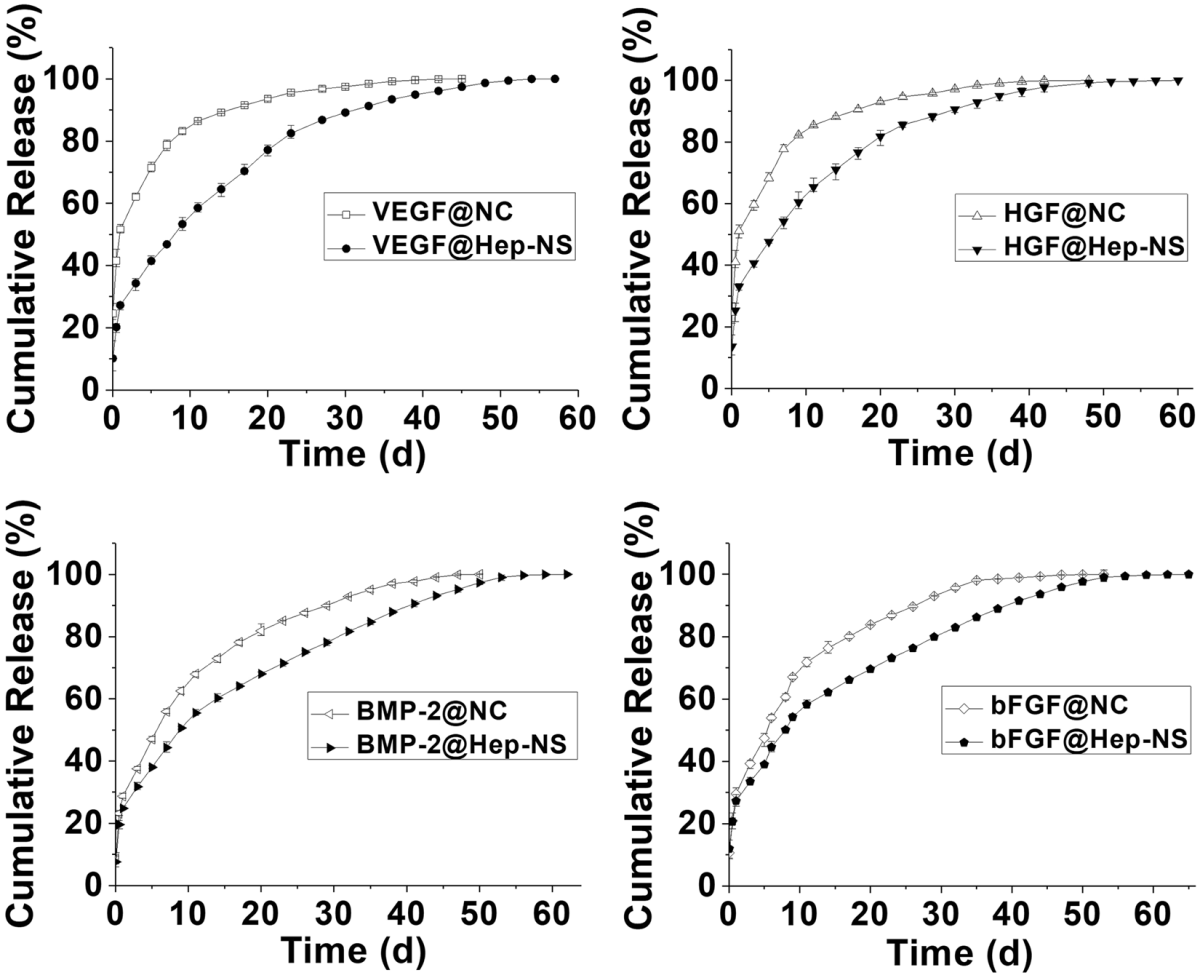
Release profiles of four different growth factors (VEGF, HGF, BMP-2, and bFGF) from the nanosponge (Hep(24)-NS) and their comparison with nano-carrier (NC) (n = 3).

In addition, four kinds of growth factor including VEGF, HGF, BMP-2, and bFGF were used to compare the release patterns between the bare Pluronic-based nano-carrier (NC) and the heparin (24 wt%)-conjugated nanosponge (Fig. 5). The growth factors were efficiently loaded into the nanosponges with over 90% encapsulation efficiency (HGF 92.4%, VEGF 92.8%, BMP-2 90.9%, and bFGF 91.8%). Heparin nanosponges (Hep-NS) showed more sustained release profiles (for a period of 60 days), than that of the bare nano-carrier (NC) without any heparin, suggesting that interactions between growth factors with heparin molecules within Hep-NS modulate their release. Among the four growth factors tested, VEGF and HGF having a relatively higher water solubility than BMP-2 and bFGF, showed faster release from the bare nano-carriers (NC), which can be explained by the even lower solubility of proteins with low water solubility in the Pluronic-based nano-carriers with a PEG-rich environment^32^. In contrast, Hep-NS resulted in sustained release profiles for all four growth factors with heparin binding affinity, regardless of their water solubility.

### Bioactivity of growth factors released from Hep-NS

**Figure 6:**
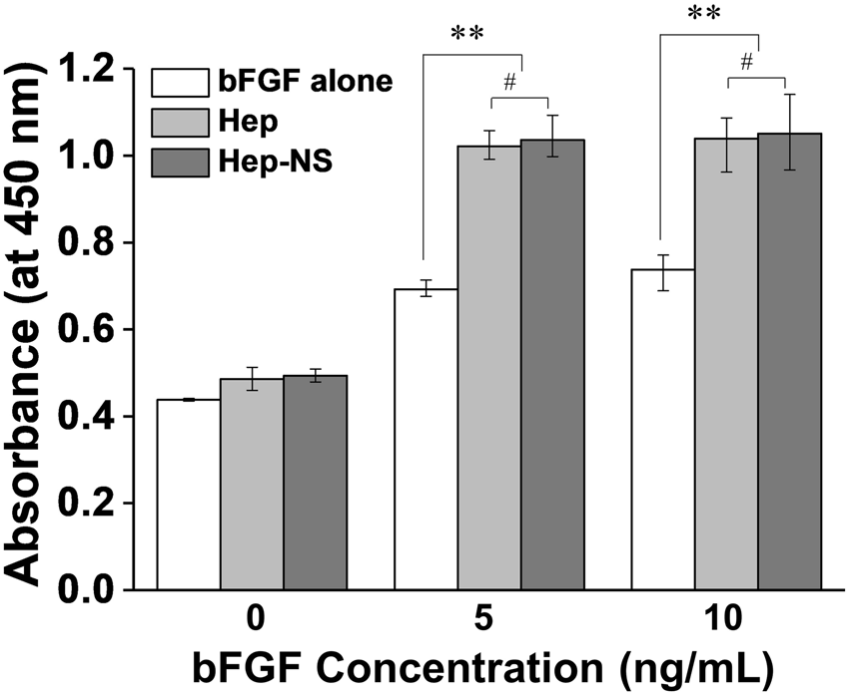
Bioactivity assay of bFGF treated with native heparin or Hep(24)-NS in Balb/c3T3 fibroblast cells (heparin concentration: 1 μg/mL). (n = 3, ^#^p > 0.05, *p < 0.05, and **p < 0.01).

To investigate the enhanced biological activity of growth factors by heparin, Balb/c3T3 fibroblast cells were cultured in bFGF containing medium with heparin or Hep-NS (24 wt%), and then analyzed by CCK-8 assay. As shown in Fig. 6, the bioactivity of bFGF with heparin (Hep) or the heparin nanosponge (Hep-NS) was well maintained at 5 and 10 ng/mL of bFGF, whereas the free bFGF resulted in much lower bioactivity compared to when heparin was used. This suggests that heparin can preserve and enhance the stability/activity of bFGF. Importantly, Hep-NS showed almost the same biological effect leading to an increase in cellular metabolic activity compared to native heparin, implying that the biological functions of heparin were well maintained in Hep-NS. In addition, the bioactivity of released bFGF from Hep-NS was well maintained up to a week (post-release) as expected (data not shown). This suggests that no denaturation of growth factors occurred during loading and release since heparin and the PEG-rich environment in the Hep-NS could synergistically preserve the stability of growth factors^32^. Therefore, the heparin nanosponge could be used as a robust and efficient vehicle for the controlled release of various growth factors, which are essential in biomedical applications; including tissue engineering, effective stem cell differentiation, and ischemia therapy amongst other uses.

In order to maintain the bioactivity of growth factors for biomedical applications, various types of delivery systems utilizing heparin have been investigated since a major goal is to deliver intact growth factors in a timely manner^1,3,9^. Previously, Jeon *et al*., have developed heparin-conjugated PLGA nanospheres for controlled long-term delivery of bFGF and demonstrated that the growth factor release profile was controlled by heparin^11^. A hyaluronate-heparin hydrogel system also showed no initial burst and long-term release of bFGF^36^. In addition, Na *et al*., reported that heparin-conjugated microspheres prepared by layer-by-layer assembly of PLGA microspheres covered with heparin/poly(_L_-lysine) nanoparticles could be used for stem-cell therapy^37^. This study showed extended immobilization of a transforming growth factor β (TGF- β) by heparin, without any loss of structural integrity and biological activity of growth factor. Heparin-conjugated polymeric micelles composed of Tetronic-PCL-heparin block copolymers have also shown improved loading of bFGF owing to specific interactions between heparin and bFGF as well as controlled release of the growth factor^38^.

Overall, the heparin nanosponge (Hep-NS) developed in this work demonstrated several favorable properties including: 1) a chemically crosslinked structure containing up to 24 wt% of heparin by a simple photo-crosslinking method, which can provide enhanced stability within an *in vivo* environment, as well as during lyophilization/resuspension, 2) temperature dependent large volumetric transition behavior, which allows for an easy loading method of active ingredients and high loading capacities, without using any toxic organic solvents and harsh conditions, 3) lower anti-coagulant activity than unmodified heparin, thus minimizing the side effects caused by heparin, 4) lower apoptosis induction (better cytocompatibility) than free heparin molecules, and 5) sustained release profiles (~2 months) of growth factors and maintenance of their bioactivity. Thus, Hep-NS is potentially a promising delivery system for the sustained release of growth factors in biomedical applications including tissue engineering.

## Methods

### Materials

Heparin (sodium salt, from porcine intestinal mucosa, molecular weight = 12,000 Da) was obtained from Cellsus Inc. (Cincinnati, OH, USA). Pluronic F 127 (PEO_100_ PPO_65_ PEO_100_, Mw = 12,600 Da) (PF 127) was donated from BASF Corp. (Seoul, Korea). 1-Ethyl-3-[3-dimethylamino]propyl]carbodiimide (EDC), 1-hydroxy-benzotriazole hydrate (HOBT), cysteamine, dithiothreitol (DTT), sodium chloride, potassium phosphate monobasic, sodium phosphate dibasic, sodium azide, Azure A chloride, and hepatocyte growth factor (HGF) were purchased from Sigma-Aldrich Corp. (St. Louis, MO, USA). 4-(2-hydroxyethoxy) phenyl-(2-hydroxy-2-propyl) ketone (Irgacure 2959) was obtained from Ciba Specialty Chemicals Inc. (Basel, Switzerland). Ellman’s reagent was purchased from Pierce (Rockford, USA). COATEST heparin kit was obtained from Chromogenix Inc. (Milano, Italy). Recombinant human vascular endothelial growth factor (VEGF), recombinant human bone morphogenetic protein 2 (BMP-2), recombinant human fibroblast growth factor basic (bFGF), and ELISA kits were obtained from PeproTech (Rocky Hill, NJ, USA). For cell experiments, DMEM (Dulbecco’s modified Eagle’s medium), fetal bovine serum (FBS), penicillin-streptomycin, and trypsin ethylenediaminetetraacetic acid (EDTA) were purchased from Gibco (Grand Island, NY, USA). The culture medium ultraMEM (containing reduced proteins) was obtained from LONZA Walkersville Inc. (Basel, Switzerland). Balb/c3T3 fibroblast cells were purchased from Korean Cell Line Bank (Seoul, Korea). All chemicals were analytical grade and were used without further purification.

### Preparation of thiol-functionalized heparin

The thiolated heparin (Hep-SH) was prepared by reacting the carboxylic group of heparin with the amine group of cysteamine, as previously reported by us^28^. Briefly, heparin was dissolved in deionized water at a concentration of 10 mg/mL. EDC, HOBt, and an excess amount of cysteamine were subsequently added (heparin:HOBT:EDC:cysteamine = 1:0.75:0.75:2) and then reacted at room temperature overnight. Next, the reaction was dialyzed by using a dialysis membrane (MWCO of 3500 Da, Spectrum lab., CA, USA) and an excess amount of DTT (10x molar ratio) was added to reduce the oxidized disulfide groups in order to obtain free thiol groups. The heparin with free thiol groups was purified by dialysis and lyophilized. The degree of thiol group substitution into heparin was measured by Ellman’s assay^28^.

### Preparation of heparin nanosponge (Hep-NS)

The heparin nanosponge was prepared by a single step photo-polymerization reaction between diacrylated Pluronic F127 (DA-PF 127) and thiolated heparin (Hep-SH)^18,19^. The DA-PF 127 solution (10 wt%, 154 μL) was diluted in deionized water (300 μL) and added into the thiolated heparin (thiolation: ~12%). The amount of thiolated heparin was varied from 0.5 mg to 7 mg to prepare the nanosponges with different amounts of heparin. Then, deionized water (1546 μL) was added into the mixture to make 0.77 wt% of DA-PF. Next, a photoinitiator (0.05 wt% of Irgacure 2959) was gently mixed with this mixture (2 mL), and the solution was UV-irradiated for 15 min at 1.3 mW/cm^2^ intensity by using an unfiltered UV lamp (VL-4.LC, 8 W, Vilber Lourmat, France). Finally, the purified nanosponges were obtained by dialysis (MWCO of 300 kDa) in de-ionized water and also by spin filtration (10000 rpm, 5 min) using Nanosep^®^ centrifugal devices (MWCO of 300 kDa, Pall Life Sciences, Ann Arbor, MI, USA). The prepared nanosponges were lyophilized and then stored at −80 °C until further use.

### Characterization of heparin nanosponges

The amount of heparin conjugated on the nanosponge was analyzed spectrophotometrically by using the cationic dye Azure A, which binds to the anionic heparin molecules^34^. The lyophilized nanosponges were resuspended in deionized water and then 20 μL of the test samples were added into a 96 well microplate. Next, 180 μL (150 μM) of Azure A solution was mixed with the sample and the mixture was incubated for 20 min at room temperature. The absorbance of the solution was measured at 595 nm by using a multiwell spectrophotometer (Spectra Max M2, Molecular Devices Inc., USA). The concentration of heparin conjugated on the nanosponge was calculated using the calibration curve for heparin.

The hydrodynamic diameters and surface charges (zeta potential) of the heparin nanosponge in de-ionized water were measured at 5, 25, or 37 °C by using an electrophoretic light scattering spectrophotometer (ELS-Z2, Otsuka Electronics Co., Japan) equipped with a laser diode light source (638 nm) and a photomultiplier tube detector (165° scattering angle). The morphologies of the nanosponges were analyzed by a transmission electron microscope (JEM-2100, JEOL, Japan) operating at 200 kV accelerating voltage. For TEM imaging, the nanosponge solutions (2 mg/mL) were mixed with 2% (w/v) phosphotungstic acid solution and incubated at 37 °C for 30 min. Then, 20 μL of the stained nanosponge suspension was dropped on the 200 mesh carbon-coated copper grid and air-dried.

### Anticoagulant bioactivity of heparin nanosponges

The anticoagulant bioactivities of the heparin nanosponges were measured and compared with free heparin and the thiolated heparin using the Coatest Heparin kit, which measures the binding affinity of heparin to antithrombin III (AT III)^27^. To prepare precursor solutions, the test samples (25 μL) in deionized water were mixed with 25 μL of AT III (0.1 unit/mL) and 200 μL of 0.1 M Tris buffer solution and then incubated for 4 min at 37 °C. Next, 25 μL of Factor Xa (FXa) was mixed with 50 μL of each precursor solutions and incubated for 30 sec at 37 °C. The mixture was subsequently mixed with 50 μL of chromogenic peptide S-2222 and incubated for 3 min at 37 °C. Finally, the reaction was stopped by adding 75 μL of acetic acid (20% v/v) and the absorbance of the solution was measured at 405 nm by using a multiwell spectrophotometer.

### *In vitro* serum stability and cytotoxicity test

To analyze the serum stability of the heparin nanosponge, the nanosponges were suspended in DMEM containing 10% FBS and 0.05% NaN_3_ and kept on a shaker at 100 rpm and 37 °C. Next, the sizes and polydispersity of the nanosponges were measured for 2 weeks.

The cytotoxicity of the nanosponges and free heparin were analyzed using Balb/c3T3 fibroblast cells. The cells (passage 2 upon use) were seeded in a 96-well tissue culture plate at a density of 1 × 10^4^ cells per well and grown in DMEM supplemented with 10% fetal bovine serum (FBS) and 1% penicillin-streptomycin for 8 h at 37 °C under 5% CO_2_. Then, the free heparin, thiolated heparin, or heparin nanosponges with concentrations ranging from 10 to 500 μg/mL (based on heparin amount) were added to the cells. After 24 h incubation, supernatants were removed and the cells were washed with PBS (pH 7.4). Next, the medium was replaced with the fresh medium containing 10-time-diluted CCK-8 (cell counting kit-8, Dojindo Laboratories, Kumamoto, Japan), and the cells were further incubated for 1 h at 37 °C. The absorbance of the colored medium was measured at 450 nm by using a multiwell spectrophotometer^39^. All measurements were done in triplicate.

### *In vitro* release profiles of growth factors from the nanosponges

To investigate whether the heparin nanosponges can effectively deliver heparin binding macromolecules, growth factors such as HGF, VEGF, BMP-2, and bFGF were used. For release experiments, each protein solution (200 ng/100 μL) was added to the lyophilized powder of nanosponge (200 μg) and was incubated at 4 °C for over 12 h, as previously reported^24,40^. Then, the whole protein-loaded nanosponge suspension was placed in a dialysis bag (cellulose ester, MWCO of 300 kDa), and subsequently immersed in 10 mL PBS containing 0.01% BSA and 0.05% NaN_3_ on a shaking rocker at 100 rpm and 37 °C. The entire release medium was replaced with a fresh one at each time point to maintain an infinite sink condition. The amount of released protein from the nanosponge was determined using ELISA. The encapsulation efficiency of proteins inside the nanosponges was determined after spin filtration at 10,000 rpm for 5 min at 37 °C and calculated by a method previously reported^40^. All measurements were performed in triplicates.

### Bioactivity analysis of growth factor with free heparin or heparin nanosponge

In order to analyze the bioactivity of bFGF, bFGF-dependent Balb/c3T3 fibroblast cells were used. The cells (passage 2 upon use) were seeded in a 96-well tissue culture plate at a density of 1 × 10^4^ cells per well and cultured in DMEM including 10% FBS and 1% antibiotics for 8 h. After incubation, the cells were washed with PBS and placed in ultraMEM (with 1% antibiotics) containing only bFGF (5 or 10 ng/mL) or bFGF mixed with free heparin or the heparin (24 wt%)-conjugated nanosponge (1 μg/mL based on heparin amount). After 40 h incubation, supernatants were removed and the cells were washed with PBS. The cell proliferation was measured by CCK-8 assay at 450 nm using a multiwell spectrophotometer^41^. All measurements were done in triplicate.

## Electronic supplementary material

Supplementary Information
