# Supplementary material for: Bioinspired Heparin Nanosponge Prepared by Photo-crosslinking for Controlled Release of Growth Factors
Source: Sci Rep. 2017 Oct 30;7:14351. doi: 10.1038/s41598-017-14040-5 (PMC5662564; doi:10.1038/s41598-017-14040-5)
Supplement: Supplementary file 1 — Supplementary Information [file 41598_2017_14040_MOESM1_ESM.pdf]

**Supplementary Information:**

# Bioinspired Heparin Nanosponge Prepared by Photo-crosslinking for Controlled Release of Growth Factors

Won Il Choi[a,1,*], Abhishek Sahu[b,1], Cristian Vilos[c,d], Nazila Kamaly[e], Seong-Min Jo[f], Jin Hyung Lee[a], Giyoong Tae[b,*]

[a] Center for Convergence Bioceramic Materials, Convergence R&D Division, Korea Institute of Ceramic Engineering and Technology, 202, Osongsaengmyeong 1-ro, Osong-eup, Heungdeok-gu, Cheongju, Chungbuk 28160, Republic of Korea.

[b] School of Materials Science and Engineering, Gwangju Institute of Science and Technology, 123 Cheomdan-gwagiro, Buk-gu, Gwangju 61005, Republic of Korea

[c] Universidad Andres Bello, Laboratory of Nanomedicine and Targeted Delivery, Center for Integrative Medicine and Innovative Science, Faculty of Medicine, Center for Bioinformatics and Integrative Biology, Faculty of Biological Sciences, Santiago, 8370071, Chile

[d] Center for the Development of Nanoscience and Nanotechnology, CEDENNA, 9170124, Santiago, Chile

[e] Technical University of Denmark, Department of Micro and Nanotechnology, DTU Nanotech, Bioinspired Nanomaterials Lab, 2800 Kgs. Lyngby, Denmark

[f] Max Planck Institute for Polymer Research, Ackermannweg 10, 55128 Mainz, Germany

* Correspondence should be addressed to: gytae@gist.ac.kr / choi830509@kicet.re.kr

[1] These authors contributed equally to this paper.

| Groups | Weeks | | |
| --- | --- | --- | --- |
| | 0 | 1 | 2 |
| **NC** | 58 ± 6 nm (0.25 ± 0.01) | 61 ± 1 nm (0.26 ± 0.02) | 60 ± 2 nm (0.27 ± 0.02) |
| **Hep(3)-NS** | 63 ± 8 nm (0.27 ± 0.02) | 65 ± 6 nm (0.28 ± 0.01) | 67 ± 6 nm (0.28 ± 0.02) |
| **Hep(6)-NS** | 63 ± 9 nm (0.28 ± 0.02) | 62 ± 3 nm (0.28 ± 0.02) | 65 ± 3 nm (0.29 ± 0.02) |
| **Hep(15)-NS** | 67 ± 4 nm (0.30 ± 0.01) | 64 ± 3 nm (0.29 ± 0.02) | 69 ± 4 nm (0.29 ± 0.03) |
| **Hep(24)-NS** | 74 ± 6 nm (0.31 ± 0.01) | 71 ± 5 nm (0.30 ± 0.01) | 73 ± 4 nm (0.29 ± 0.02) |

**Supplementary Table S1**. *In vitro* serum stability of the nano-carrier (NC) and heparin nanosponge (Hep-NS). (n=3)

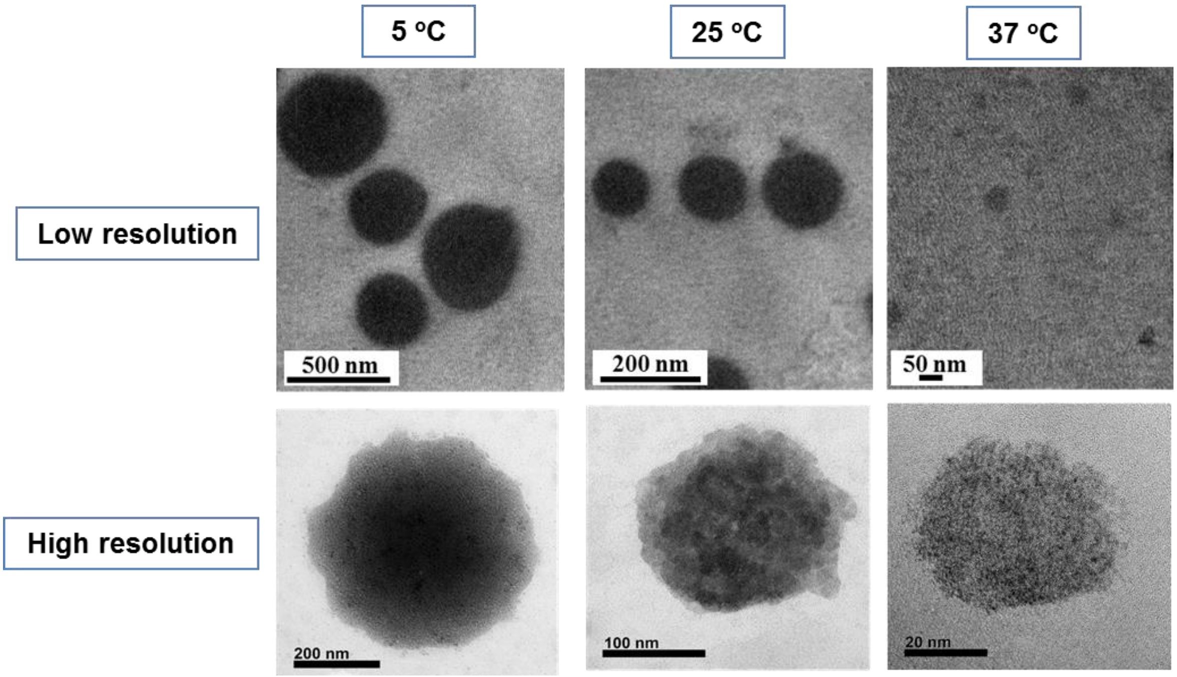


**Supplementary Figure S1**. TEM images of Hep(24)-NS samples pre-equilibrated at 5 °C, 25 °C, and 37 °C.
